# Synthesis and Characterization of Highly Sensitive Hydrogen (H_2_) Sensing Device Based on Ag Doped SnO_2_ Nanospheres

**DOI:** 10.3390/ma11040492

**Published:** 2018-03-26

**Authors:** Zhaorui Lu, Qu Zhou, Lingna Xu, Yingang Gui, Zhongyong Zhao, Chao Tang, Weigen Chen

**Affiliations:** 1 College of Engineering and Technology, Southwest University, Chongqing 400715, China; lu_zhaorui@163.com (Z.L.); lingnaxu@cqu.edu.cn (L.X.); yinganggui@swu.edu.cn (Y.G.); zhaozy1988@swu.edu.cn (Z.Z.); tangchao_1981@163.com (C.T.); 2 State Key Laboratory of Power Transmission Equipment & System Security and New Technology, Chongqing University, Chongqing 400030, China; weigench@cqu.edu.cn

**Keywords:** Ag doping, SnO_2_ nanospheres, synthesis and characterization, H_2_ sensing device

## Abstract

In this paper, pure and Ag-doped SnO_2_ nanospheres were synthesized by hydrothermal method and characterized via X-ray powder diffraction (XRD), field emission scanning electron microscopy (FESEM), energy dispersive spectroscopy (EDS), and X-ray photoelectron spectra (XPS), respectively. The gas sensing performance of the pure, 1 at.%, 3 at.%, and 5 at.% Ag-doped SnO_2_ sensing devices toward hydrogen (H_2_) were systematically evaluated. The results indicated that compared with pure SnO_2_ nanospheres, Ag-doped SnO_2_ nanospheres could not only decrease the optimum working temperature but also significantly improve H_2_ sensing such as higher gas response and faster response-recovery. Among all the samples, the 3 at.% Ag-doped SnO_2_ showed the highest response 39 to 100 μL/L H_2_ at 300 °C. Moreover, its gas sensing mechanism was discussed, and the results will provide reference and theoretical guidance for the development of high-performance SnO_2_-based H_2_ sensing devices.

## 1. Introduction

Hydrogen (H_2_), as one of the cleanest, most efficient, abundant and renewable energies, has attracted worldwide attention in the past few decades [1,2,3,4]. It has extensive applications in fuel cells, nuclear power plants, industry, petroleum refining and aerospace [5,6]. However, H_2_ will be easy to explode in a wide range of concentration (4–75%) with low ignition energy (0.02 mJ) [7]. Therefore, in industrial process control and applications, it is necessary to develop rapid and accurate sensor to detect the leakage of hydrogen storage, transportation and usage [8,9].

Numerous types of H_2_ sensors based on different principles like the resistive type, thermoelectric type and optical fiber have been reported [10,11,12,13]. Among these, metal-oxide semiconductor (MOS) sensors take a special position [14,15,16]. In particular, SnO_2_ is particularly remarkable due to its high electron mobility, low cost and good chemical properties [17]. Metal doping is one of the most effective approaches to enhance the gas-sensing performances [18,19,20]. For example, Wang et al. studied Au-loaded SnO_2_ gas sensor with several dopants concentrations, and showed that 4.0 at.% Au-loaded SnO_2_ exhibited the highest response value 25 toward 100 ppm H_2_ at 250 °C [17]. Dae-Hyun Baek et al. reported MoS_2_ gas sensor functionalized by Pd successfully detected hydrogen gas diluted by air at room temperature [18]. Liu et al. reported 1 wt % Co-doped SnO_2_ nanofibers, and the measured results exhibited the highest response to 24 along with a short response and recovery time (2 s, 3 s) toward 100 ppm H_2_ at 330 °C [21]. Mehar Bhatnagar et al. investigated the incorporation of C in SnO_2_ nanoparticles, and excellent selectivity towards H_2_ and ethanol in the low temperature range [22]. Some works have been reported that researchers use Ag as a catalytic dopant on the surface of SnO_2_ to improve its performance of gas sensing. For example, Wu et al. reported that the Ag-doped SnO_2_ sensor response was 2.24, and the response-recovery time were as short as 34 s and 68 s, respectively in an ethanol gas detection system [23]. Based on the density functional theory and the first-principles, Jin et al. built the pure and Ag-doped SnO_2_ models and gas adsorption models, and interesting calculations were conducted [24]. However, the influence of the ratio of Ag dopant in SnO_2_ on detecting H_2_ has not been reported. We further focused on the morphology of SnO_2_ nanostructures and the ratio of Ag dopant in SnO_2_ for the purpose of H_2_ detection.

In this work, we have successfully synthesized pure and Ag-doped SnO_2_ nanospheres materials and systematically researched their gas sensing performances to H_2_. The effects of Ag doping on SnO_2_-based H_2_ detection response were analyzed, which showed a significant dependence of H_2_ sensing performance on Ag concentration. The results indicated that the introducing of moderate Ag plays an important role in improving the sensing performances of pure SnO_2_ nanospheres to H_2_, in terms of lower optimal working temperature, higher gas response and shorter response-recovery time. Moreover, its gas sensing mechanism was also discussed in detail. 

## 2. Materials and Methods 

### 2.1. Materials

All raw chemicals were analytical graded and purchased from Chongqing Chuandong Chemical Reagent Co., Ltd. and were used as received without any further purification.

### 2.2. Synthesis of the Sensing Materials

Undoped and Ag-doped SnO_2_ nanosphere gas sensitive materials were prepared using the simple hydrothermal method. First, 2.67 g of sodium stannate (Na_2_SnO_3_·3H_2_O), 2.4 g of sodium hydroxide (NaOH) and 0.8 g of citric acid (C_6_H_8_O_7_·H_2_O) were dissolved into the binary solution containing 80 mL of anhydrous ethanol and 80 mL of deionized water. Next, different masses of AgNO_3_·5H_2_O (0 g, 0.026 g, 0.078 g, 0.13 g, corresponding to Ag/Sn ratios of 0, 1, 3 and 5 at.%) was added to the above mentioned solution. Then, the mixture was continuously stirred at constant temperature for 30 min to prepare a homogeneous precursor solution. The mixed precursor solution was transferred into a 200 mL Teflon lined stainless steel autoclave and heated at 180 °C for 20 h. After the sample was cooled to room temperature, the sample was washed four times with deionized water and absolute ethanol to remove impurity respectively. The samples were finally obtained after air dried at 80 °C for 24 h.

### 2.3. Characterization of the Sensing Materials

The phase of the resultant powders were investigated by X-ray diffraction (XRD, D/Max-1200X, Rigaku, Tokyo, Japan) with Cu-Kα radiation (λ = 1.54178 Å), and the scanning speed was 0.02° s^−1^ for 2θ in the range of 20°–80°. The morphology of resultant powders was performed with field emission scanning electron microscopy (FESEM, JSM-6700F, JEOL, Tokyo, Japan). The elemental composition of the obtained samples was analyzed using energy dispersive spectroscopy (EDS, Oxford INCA 250, JEOL, Tokyo, Japan) and X-ray photoelectron spectroscopy (XPS, KRATOS X SAM800, Kratos, Manchester, Kingdom). 

### 2.4. Fabrication and Measurements of the Sensing Devices 

**Figure 1:**
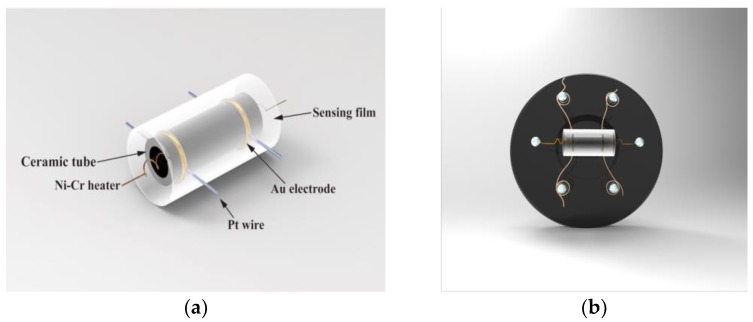
Schematic diagram of (**a**) ceramic tube and (**b**) the indirect-heating sensor.

In the present experiment, sensing devices were fabricated with the side heated structure. The as-prepared powders were mixed with suitable amount of anhydrous ethanol and deionized water (with 8:1:1 ratio) to form a homogeneous paste, which was coated evenly onto a prefabricated alumina tube attached with a pair of gold electrodes and platinum wires to form a film, then dried at room temperature and subsequently calcined at 500 °C for 4 h (Figure 1a). Next a Ni–Cr heating wire was inserted in the tube to form an inside heated sensing device (Figure 1b). Finally, the sensing devices were placed on the aging instrument of the side heat sensor at 120 °C for 10 days to improve the stability of the components. The gas sensing properties of the fabricated sensing devices were measured by a CGS-8 (Chemical gas sensor-8, Beijing Elite Tech Co., Ltd., Beijing, China) intelligent gas sensing analysis system (Beijing Elite Tech Co., Ltd., Beijing, China). The gas sensor response was defined as *R*_a_/*R*_g_, where *R*_a_ and *R*_g_ were the resistance of the sensor in air and in the test gas, respectively [25]. 

## 3. Results and Discussion 

### 3.1. Materials Characterization

**Figure 2:**
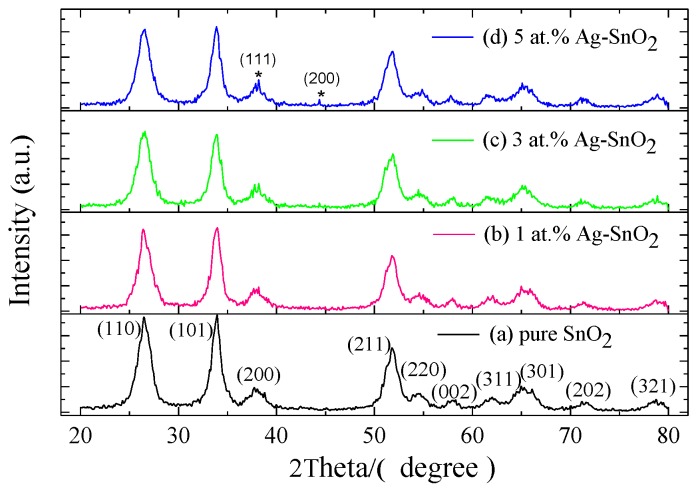
XRD patterns of pure and 1 at.%, 3 at.%, 5 at.% Ag-doped SnO_2_.

Figure 2 shows the XRD patterns of pure and 1 at.%, 3 at.%, 5 at.% Ag-doped SnO_2_ nanospheres. It can be found that the XRD patterns of the samples are smooth and the shape of the peak is sharp, indicating that the prepared samples have well developed to crystal grains and showed good crystallization performance. As shown in Figure 2a, all the diffraction peaks can be readily indexed to the tetragonal phase of rutile SnO_2_ structure, good agreement with the reported values (JCPDS card No. 41-1445) without any other phase detected, indicating that pure SnO_2_ has been obtained [26]. The XRD patterns of 1 and 3 at.% Ag-doped SnO_2_ shows almost no change compared with that of the pure SnO_2_ products, which may be due to the poor amount of Ag in the Ag-SnO_2_ nanospheres [27]. Moreover, the diffraction peaks labeled as (111) and (200) in Figure 2d is observed, which can be indexed to the face centered cubic phase of Ag nanoparticles (JCPDS card No. 04-0783).

The crystallite sizes (*d*) of the pure SnO_2_ and 5 at.% Ag-doped SnO_2_ nanomaterials were measured by the well-known Debye-Scherer equation (Equation (1)).

(1) $$d=\frac{0.89\lambda }{\beta \cdot \cos\theta }$$

where λ is the X-ray wavelength and has a value of 1.542 Å, θ is the Bragg diffraction angle and β is the full width at half maximum (FWHM). For estimating the crystallite size of pure and Ag-doped SnO_2_ nanomaterials accurately, the three most intense peaks corresponding to (110), (101) and (211) diffraction planes were calculated and shown in Table 1. The average crystallite size of the pure and 5 at.% Ag-doped SnO_2_ were found to be 4.38 nm and 4.07 nm, respectively.

**Figure 3:**
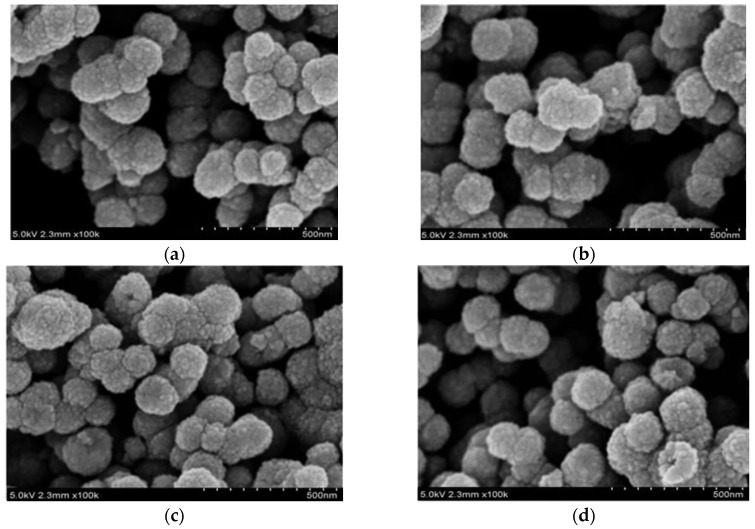
FESEM images of (**a**) pure; (**b**) 1 at.%; (**c**) 3 at.%; and (**d**) 5 at.% Ag doped SnO_2_.

As shown in Figure 3, the size distribution and the morphology of the as-prepared pristine and Ag-doped samples were analyzed by FESEM. All the samples are nearly spherical structure and the diameters of all microspheres are in the scope of 80 to 120 nm. Moreover, good dispersion of all samples is also observed. The FESEM images indicate that the doping does not change the morphology and surface structure of SnO_2_ samples.

**Figure 4:**
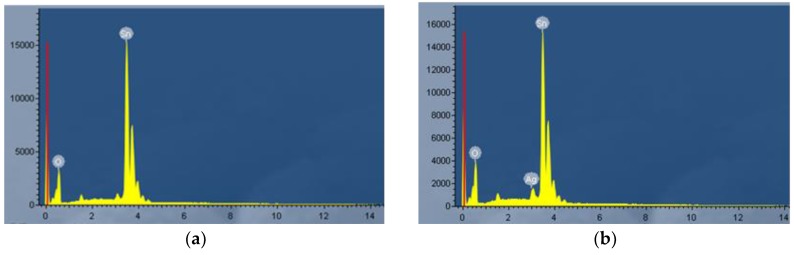
EDS spectra of (**a**) pure (**b**) 3 at.% Ag-doped SnO_2_ nanospheres.

In order to check whether metallic Ag was successfully doped into the SnO_2_ nanomaterials, EDS measurements were performed, and the EDS spectra of the pure and 3 at.% Ag-doped SnO_2_ are shown in Figure 4a,b, respectively. As shown in Figure 4a, only Sn and O peaks are observed for SnO_2_, indicating that as-prepared SnO_2_ is of high purity. The strong signals of elemental Sn, O and a weak signal of Ag are detected in Figure 4b, which indicates that the prepared SnO_2_ nanomaterials are successfully doped with Ag and the atomic percent of Ag is calculated to be about 2.98 at.%.

**Figure 5:**
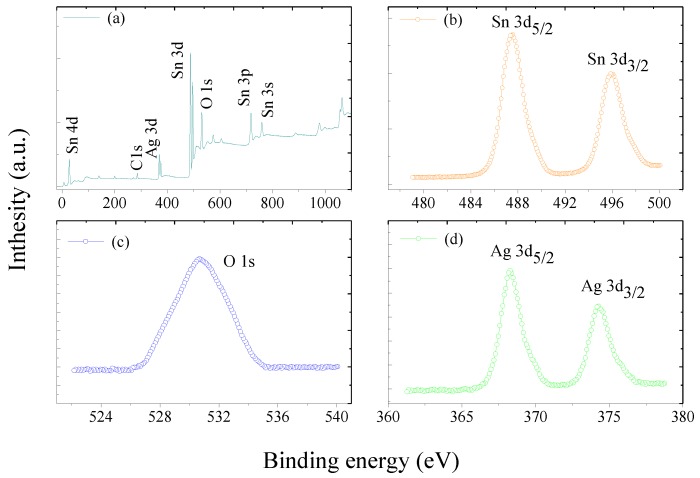
XPS survey spectra of 3 at.% Ag-doped SnO_2_ nanospheres (**a**) full; (**b**) Sn 3d; (**c**) O 1s; (**d**) Ag 3d.

For further analyzing the elemental composition of the obtained samples and the valence of each element, XPS tests were investigated. The XPS spectrum of the synthesized 3 at.% Ag-doped SnO_2_ nanospheres is represented in Figure 5a, where spectra from Sn, O and Ag elements are observed and the Ag atom concentrations in the composites is 2.98 at.%. In order to further investigate the existence state of Sn, O and Ag in the prepared materials, the enlarged XPS survey spectra of Sn 3d, O 1s, Ag 3d are showed in Figure 5b–d, respectively. Figure 5b,c show the binding energy of Sn 3d_5/2_, Sn 3d_3/2_ and O 1s are 486.85 eV, 495.35 eV and 530.59 eV respectively, which are the confirmatory peaks for a Sn^4+^ and O^2−^ ions of SnO_2_ and in good accordance with the standard parameter values [28]. As shown in Figure 5d, the Ag 3d spectrum exhibits doublets of Ag 3d_5/2_ and Ag 3d_3/2_ at 368.21 eV and 374.19 eV, which correspond to the state of metallic silver [29].

### 3.2. Hydrogen Gas Sensing Studies 

**Figure 6:**
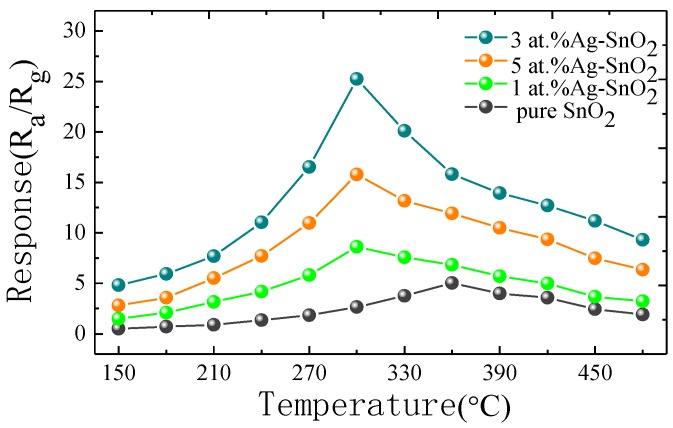
Gas responses of pure, 1 at.%, 3 at.% and 5 at.% Ag-doped SnO_2_ based sensor to 50 μL/L H_2_ at different working temperature.

In order to investigate the optimal operating temperature of the fabricated sensors to detect H_2_, the gas sensing responses of the pure and Ag-doped SnO_2_ towards 50 μL/L of H_2_ gas were measured respectively, with operating temperatures ranging from 150 °C to 480 °C. As shown in Figure 6, with the increase of the temperature, the sensing response of all the prepared sensors increases at first and attains a maximum value at a particular temperature. Then it decreases with further increase of temperature. The operating temperature could be taken as a balance between two processes adsorption and desorption [30]. When the temperature is larger than the particular value, the adsorption of oxygen becomes increasingly inefficient and the active oxygen species reduces in quantity, so the gas response begins to decline. When exposed to 50 μL/L H_2_, the measured optimal operating temperature of the 1 at.%, 3 at.% and 5 at.% Ag-doped SnO_2_ sensors is 300 °C with the corresponding response of 8.61, 25.25 and 15.78, respectively. The notable difference of responses between pure and Ag-doped SnO_2_ can be attributed to the catalytic activity of Ag [31]. As comparison, the response of the fabricated pure SnO_2_ for 50 μL/L H_2_ at operating temperature of 360 °C is 5.04, which is lower than those of Ag-doped SnO_2_. It could be explained that Ag doping influences the shift of optimal operating temperature towards lower temperature due to a decrease in the band gap [27]. The 3 at.% Ag-doped SnO_2_ sensor exhibits the highest H_2_ gas response among the four sensors. The decrease of response for the doped sensor above 3 at.% Ag is observed, possibly due to the reduction of active sites associated with the agglomeration of Ag nanoparticles [32].

**Figure 7:**
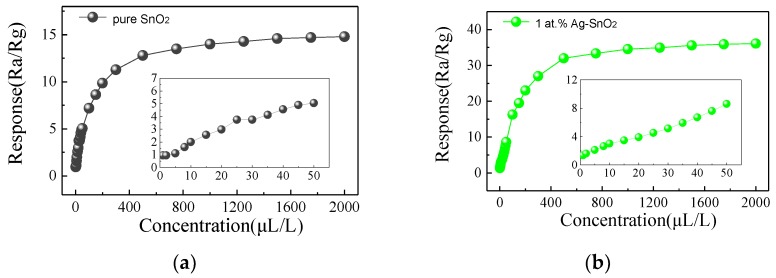

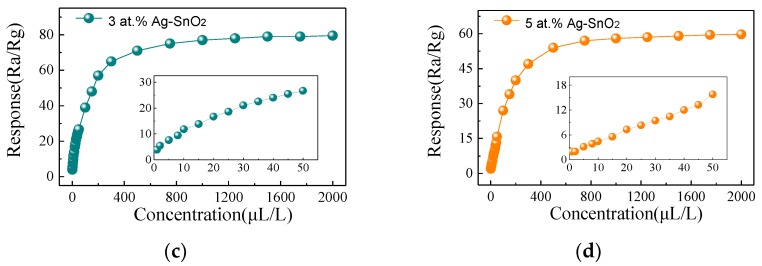
Gas responses of pure,1 at.%,3 at.% and 5 at.% Ag-doped SnO_2_ based sensor versus different concentration of H_2_ under their optimum operating temperature. (**a**) Pure; (**b**) 1 at.% Ag-doped SnO_2_; (**c**) 3 at.% Ag-doped SnO_2_; (**d**) 5 at.% Ag-doped SnO_2_.

In order to explore the relation between response and the concentration of H_2_, experiments of responses of pure and Ag-doped SnO_2_ sensors to various concentration of H_2_ (from 1 μL/L to 2000 μL/L) are conducted at their own optimum operating temperature and the results are presented in Figure 7. It is apparent that the response of above samples increases rapidly within 1–500 μL/L H_2_ gas concentration and then slows down so that it converges to a constant. It can be known that the sensors are almost at saturation when the concentration of H_2_ above 1000 μL/L. Compared with the other three sensors, the 3 at.% Ag-doped SnO_2_ sensor exhibits the highest response towards certain concentration of H_2_ gas, which might be attributed to the appropriate incorporated of Ag nanoparticles [33]. Obviously, the gas response exhibits a linear relationship with gas concentration when the latter ranges from 1 to 50 μL/L (inset of Figure 7), indicating that the sensors suit well for low concentration detection.

**Figure 8:**
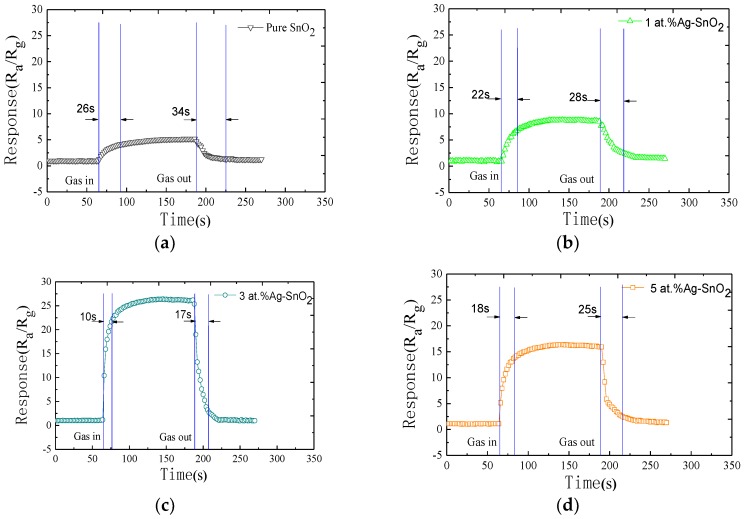
Response-recovery curves of the as-prepared sensors to 50 µL/L H_2_ at their own optimum operating temperature: (**a**) pure SnO_2_ sensor; (**b**) 1 at.% Ag-doped SnO_2_ sensor; (**c**) 3 at.% Ag-doped SnO_2_ sensor; (**d**) 5 at.% Ag-doped SnO_2_ sensor.

It is significant that the sensor has a swift response and rapid recovery time in the real time fast changing environment. Response and recovery times of gas sensors are usually defined as the time required while reaching 90% of the final resistance in the case of the process of adsorption and desorption, respectively. Figure 8 shows response-recovery behavior of the pure, 1 at.%, 3 at.% and 5 at.% Ag-doped SnO_2_ sensors to 50 µL/L of H_2_ gas at their own optimum operating temperature. The response and recovery times for the pure, 1 at.%, 3 at.% and 5 at.% Ag-doped SnO_2_ sensors of 50 µL/L H_2_ are 26–34 s, 22–28 s, 10–17 s and 18–25 s, respectively. It proves that the 3 at.% Ag-doped SnO_2_ sensor has better sensing performance.

**Figure 9:**
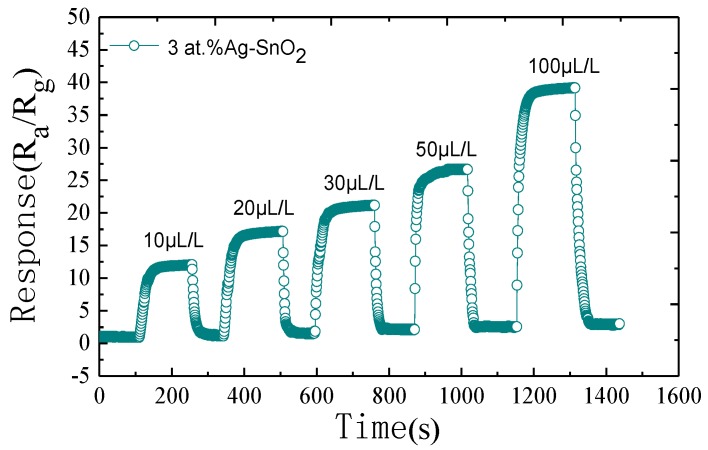
Dynamic response-recovery curve of the 3 at.% Ag-doped SnO_2_ sensor to H_2_ in a range of 10–100 µL/L under its optimum working temperature.

Figure 9 depicts the response-recovery curve of the 3 at.% Ag-doped SnO_2_ sensor to H_2_ in a range of 10–100 µL/L under its optimum working temperature. Clearly, the as-prepared sensor exhibit a rapid response-recovery times, and its can recover with the nearly initial values after many cycles between exposure to H_2_ and air.

**Figure 10:**
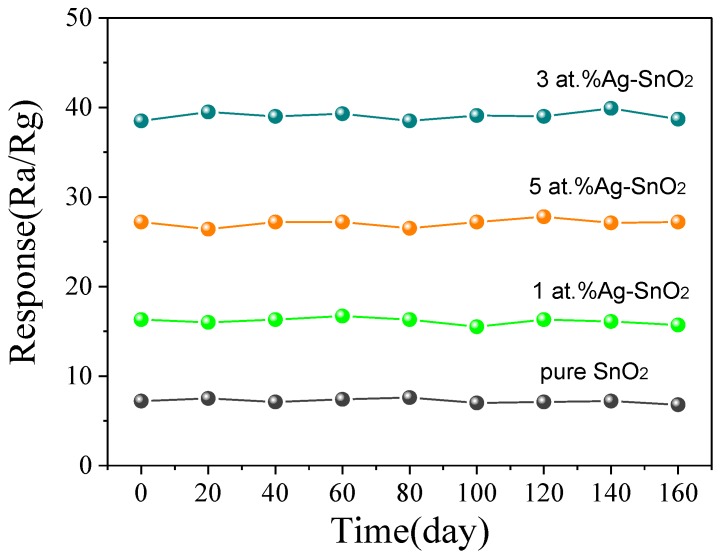
The long-term stability of pure, 1 at.%, 3 at.% and 5 at.% Ag-doped SnO_2_ to 100 µL/L H_2_ at their optimum temperatures.

The stability of the fabricated pure, 1 at.%, 3 at.% and 5 at.% Ag-doped SnO_2_ to 100 µL/L H_2_ at their optimum temperatures was investigated every 20 days for total 160 days. Figure 10 shows the response variation curves and it is obvious that the responses changed very slightly. Therefore, a good stability of the sensors was conformed.

### 3.3. Hydrogen Sensing Mechanism 

**Figure 11:**
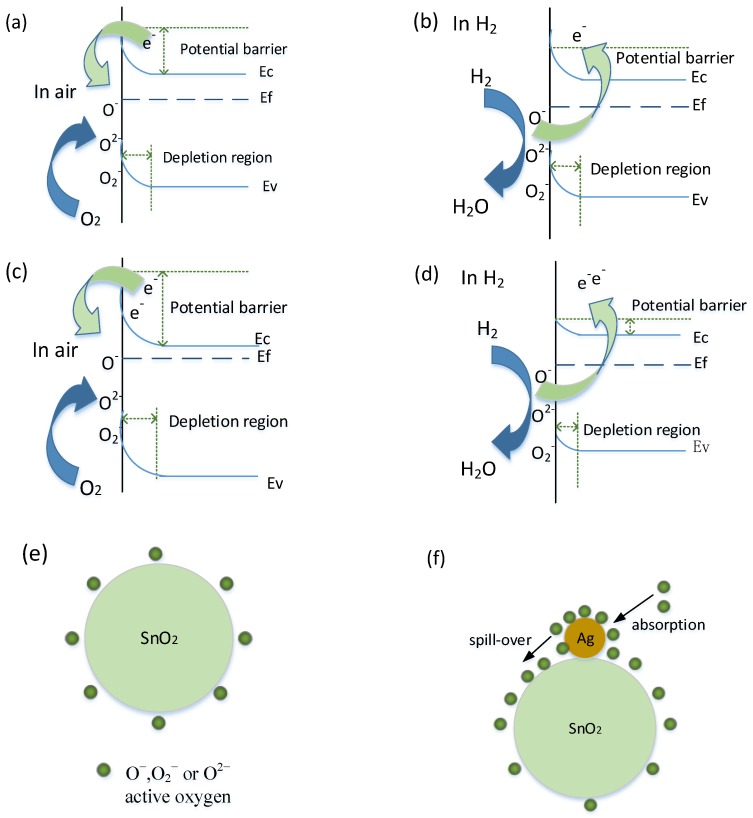
The sensing mechanism of pure and Ag-doped SnO_2_: (**a**) pure SnO_2_ in air; (**b**) pure SnO_2_ in H_2_ gas; (**c**) Ag-doped SnO_2_ in air; (**d**) Ag-doped SnO_2_ in H_2_ gas; (**e**) active oxygen distribution of pure SnO_2_; (**f**) active oxygen distribution of Ag-doped SnO_2_.

It is well known that the gas sensing mechanism of SnO_2_ gas sensor belongs to the surface-controlled type, and the gas sensing performance is highly dependent upon the surface reactions between the target gas and adsorbed oxygen species on the surface area of SnO_2_ [34]. Figure 11 illustrates the schematic diagram for sensing mechanism of the H_2_ sensors based on pure and Ag-doped SnO_2_ nanomaterials, where Ef, Ec and Ev denote Fermi level, conduction band and valence band, respectively. When pure SnO_2_ gas sensor is exposed to air (Figure 11a), oxygen molecules can be adsorbed on the sensor surface and capture electrons from the conduction band of SnO_2_ to generate chemisorbed oxygen species (O^−^,O_2_^−^ and O^2^^−^), which results in a depletion layer on the surface and the decreasing electrical conductivity of sensing materials [35,36,37]. When SnO_2_ sensing materials are exposed to H_2_ (Figure 11b), H_2_ gas interacts with the adsorbed oxygen species, and then the trapped electrons are released back into the conduction band of SnO_2_, thereby increasing its conductivity.

Compared with the pure SnO_2_, the Ag-doped SnO_2_ composite sensing materials exhibit enhanced gas sensing properties, which might be ascribed to the following aspects. Firstly, as we know, the work function of Ag (4.72 eV) is higher than that of SnO_2_ (4.60 eV), and the Schottky junctions would form between Ag and SnO_2_, which causes electrons transfer from SnO_2_ to Ag [38]. Thus, a depletion region would be formed in SnO_2_ near the interface of Ag and SnO_2_ [39,40]. Secondly, Ag nanoparticles can act as active site to reduce the reaction barrier between H_2_ and the adsorbed oxygen species due to its good catalytic ability, which results in a further extend in width of the depleted layer [23]. In addition, Ag nanoparticle has a tendency to form Ag_2_O in the air and Ag_2_O is a kind of p type semiconductor that will further intense electron depletion layer on the sensors surface [27,41]. Meanwhile, as shown in Figure 11e,f, the presence of Ag nanoparticle favors the gas sensing response by the process of chemical sensitization, catalytic oxidation (spill-over effect), resulting in increasing the quantities of active oxygen species on the surface of the Ag-doping SnO_2_ nanocomposite [34,42]. Thus, the resistance of the Ag-doped SnO_2_ gas sensors become significantly larger than that of pure SnO_2_ sensor in air (Figure 11c). When the sensor is exposed to H_2_ (Figure 11d), the thick electron depletion layer would decrease sharply to a thin layer by the reaction of H_2_ and adsorbed oxygen species, leading to a significantly enhanced gas response [43,44]. Table 2 compares the H_2_ sensing performances of the fabricated 3 at.% Ag-doped SnO_2_ nanospheres based sensor with the different sensors reported in the literature. The presented Ag-SnO_2_ nanospheres exhibited comparatively better gas response with low response and recovery times.

## 4. Conclusions

In summary, Ag-doped SnO_2_ nanospheres with different atomic percent (pure, 1 at.%, 3 at.% and 5 at.%) have been successfully synthesized by a hydrothermal process for the fabrication of highly sensitive H_2_ sensing devices. The crystalline structures and morphologies of as-prepared nanomaterials were characterized via XRD and FESEM, proving the microspheres nanostructures. The EDX and XPS patterns verified the element components and valences, and the standard peaks of metallic silver were obviously observed. The gas sensing properties of prepared nanomaterials have been investigated. The main characteristics of the fabricated Ag-doped SnO_2_ sensor are wide range of H_2_ response(1–2000 μL/L), lower temperature operation, quick response recovery times, as well as good stability over time. Gas sensing results demonstrate that the obtained 3 at.% Ag-doped SnO_2_ sensor shows the best hydrogen sensing performance at 300 °C, suggesting that the moderate Ag-doped SnO_2_ sensor is highly promising for H_2_ sensing application.

## Figures and Tables

**Table 1:** Different XRD parameters for the determination of crystallite sizes.

Nanomaterials	(hkl)	2Theta (°)	FWHM (β)	Crystallite Size (nm)
Pure SnO_2_	(110)	26.48	2.1	3.85
	(101)	33.98	1.75	4.69
	(211)	51.78	1.9	4.60
5 at.% Ag-SnO_2_	(110)	26.58	2.3	3.51
	(101)	33.88	1.95	4.22
	(211)	51.88	1.95	4.48

**Table 2:** Summary of the H_2_ gas sensing performances of different gas sensor materials.

Sensing Material	Concentration	Temp. (°C)	Response	Response Formula	Response Time (s)	Recoverytime (s)	Ref.
Pd-SnO_2_/MoS_2_	5000 ppm	R.T.	18%	(*R*_a_ − *R*_g_)/*R*_a_ × 100%	30	19	[14]
Pt/SnO_2_	500 ppm	110	168	(*R*_a_ − *R*_g_)/*R*_g_	<6	57	[38]
WO_3_-SnO_2_	2000 ppm	225	52.39	*R*_a_/*R*_g_	6.6	-	[45]
Au/SnO_2_	5000 ppm	400	50	*R*_a_/*R*_g_	25	170	[46]
CeO_2_-SnO_2_	0.5 ppm	300	−82	*R*_a_/*R*_g_	~50	~30	[47]
Ag/SnO_2_	50 μL/L	300	25.25	*R*_a_/*R*_g_	10	17	This work
